# Acute liver failure and secondary TMA-like phenomenon following SARS-CoV-2 and *Mycoplasma pneumoniae* co-detection in a child: a case report

**DOI:** 10.3389/fmed.2026.1849049

**Published:** 2026-05-28

**Authors:** Qian Liu, Ting Kang, Wenjuan Zeng, Yuan Long

**Affiliations:** Western Emergency Department, Wuhan Children's Hospital, Tongji Medical College, Huazhong University of Science & Technology, Wuhan, Hubei, China

**Keywords:** acute liver failure, co-detection, *Mycoplasma pneumoniae*, SARS-CoV-2, secondary TMA-like phenomenon

## Abstract

**Background:**

In the post-pandemic era, the co-detection of SARS-CoV-2 and *Mycoplasma pneumoniae* (*M. pneumoniae*) is increasingly recognized in children, but its full clinical spectrum remains poorly characterized.

**Case report:**

We describe a previously healthy 20-month-old girl who presented with sepsis, acute liver failure (ALT, 17,134 U/L), and extreme hyperinflammation (ferritin, 10,929.6 ng/mL; interleukin-6 [IL-6], 74 pg/ml) associated with SARS-CoV-2 and *M. pneumoniae* co-detection. Despite initial improvement in the liver function with glucocorticoid therapy, she subsequently developed a secondary thrombotic microangiopathy (TMA)-like phenomenon characterized by severe thrombocytopenia (52 × 10^9^/L), microangiopathic hemolysis, and acute kidney injury (creatinine, 181.5 μmol/L). A comprehensive etiological workup, including negative complement gene testing (whole-exome sequencing, whole-genome sequencing, and copy number variation analysis), supported a diagnosis of severe infection-associated secondary TMA-like phenomenon. The patient recovered after comprehensive supportive care, antimicrobial therapy, glucocorticoids, and prophylactic anticoagulation.

**Conclusion:**

This case delineates a clinical sequence from acute liver failure to a secondary TMA-like phenomenon associated with SARS-CoV-2 and *M. pneumoniae* co-detection. The transient C3 consumption with spontaneous recovery, together with negative complement gene testing, was documented. This report highlights the importance of vigilant monitoring for a secondary TMA-like phenomenon in children with severe hyperinflammation and co-detection of viral and bacterial pathogens.

## Introduction

In the post-pandemic era, SARS-CoV-2 is recognized not only as a respiratory pathogen but also as a cause of systemic illness (1). In children, most cases are mild, but a minority develop multisystem inflammatory syndrome (MIS-C) or severe organ dysfunction (2, 3). Concurrently, *Mycoplasma pneumoniae* (*M. pneumoniae*), a major atypical pathogen of community-acquired pneumonia, has also been reported to trigger immune-mediated liver injury (4), hemolytic anemia, and even thrombotic microangiopathy (TMA) (5, 6). Several studies have reported the co-detection of these two pathogens. Marino et al. have found that among 5,867 children with COVID-19, 9.1% had a co-detection with *M. pneumoniae* (7). Similarly, Korneenko et al. have reported that 12.4% of children hospitalized for *M. pneumoniae* had a co-detection with SARS-CoV-2 (8). The majority of these co-detections present with respiratory symptoms, with only a few, such as the case reported by Grama et al., describing extrapulmonary manifestations (9). To the best of our knowledge, the sequential occurrence of acute liver failure followed by the secondary TMA-like phenomenon after co-detection has not been previously reported. In this study, we report the case of a 20-month-old girl with a SARS-CoV-2 and *M. pneumoniae* co-detection who developed acute liver failure followed by a secondary TMA-like phenomenon. This case highlights the diagnostic challenge of a secondary TMA-like phenomenon in severe pediatric co-detections.

## Case report

**Figure 1:**
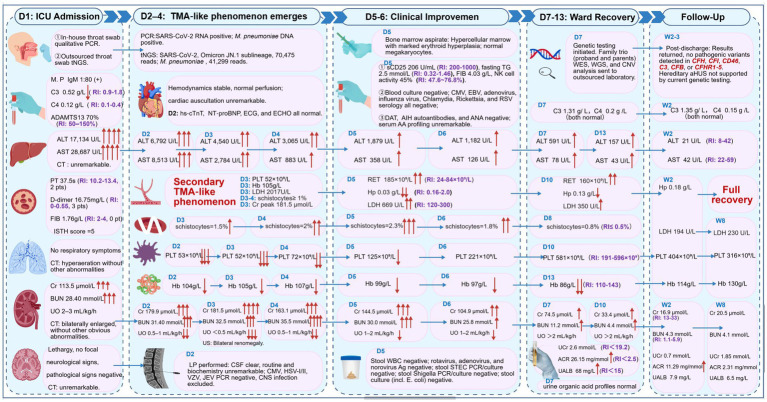
Timeline of disease evolution and multisystem assessment. PCR, polymerase chain reaction; tNGS, targeted next-generation sequencing; *M. pneumoniae*, *Mycoplasma pneumoniae*; C3, complement component 3; C4, complement component 4; RI, reference interval; ALT, alanine aminotransferase; AST, aspartate aminotransferase; CT, computed tomography; PT, prothrombin time; FIB, fibrinogen; ISTH, International Society on Thrombosis and Haemostasis; DIC, disseminated intravascular coagulation; Cr, creatinine; BUN, blood urea nitrogen; UO, urine output; hs-cTnT, high-sensitivity cardiac troponin T; NT-proBNP, N-terminal pro-B-type natriuretic peptide; ECG, electrocardiogram; ECHO, echocardiography; EF, ejection fraction; TMA, thrombotic microangiopathy; Hp, haptoglobin; Ret, reticulocyte; PLT, platelet count; Hb, hemoglobin; LDH, lactate dehydrogenase; sCD25, soluble interleukin-2 receptor alpha; TG, triglycerides; NK, natural killer; CMV, cytomegalovirus; EBV, Epstein–Barr virus; RSV, respiratory syncytial virus; DAT, direct antiglobulin test; AIH, autoimmune hepatitis; ANA, antinuclear antibody; AA, amino acid; WES, whole-exome sequencing; WGS, whole-genome sequencing; CNV, copy number variation; aHUS, atypical hemolytic uremic syndrome; CSF, cerebrospinal fluid; UCr, urinary creatinine; ACR, urinary albumin-to-creatinine ratio; UALB, urinary microalbumin. Created with http://BioGDP.com.

A previously healthy 20-month-old girl presented to the emergency department with a 2-day history of high fever, frequent vomiting, and diarrhea. On admission, she was acutely ill, lethargic, and poorly responsive, with cold extremities and borderline hypotension (90/48 mmHg). Initial laboratory investigations (Table 1) revealed severe metabolic acidosis (pH, 7.16; lactate, 5.0 mmol/L; and base excess, −17.6 mmol/L), acute liver failure (ALT, 17,134 U/L), markedly elevated D-dimer, renal dysfunction, hypoglycemia (1.4 mmol/L), and a cytokine storm pattern of inflammatory markers (ferritin, 10,929.6 ng/mL; IL-6, 74 pg/ml), with a normal blood ammonia level (1 μmol/L; reference interval [RI], 18–72 μmol/L). Computed tomography (CT) imaging showed no intracranial abnormality, hyperaerated lungs without other findings, and bilaterally enlarged kidneys with a normal-appearing liver. The clinical picture was consistent with sepsis, disseminated intravascular coagulation, and acute liver failure. The child was immediately transferred to the pediatric intensive care unit. Blood drawn immediately after admission showed normal ADAMTS13 activity, normal C4, and decreased C3 (Figure 1). A microbiological workup (outsourced throat swab tNGS and in-house qualitative polymerase chain reaction [PCR]) confirmed the co-detection of SARS-CoV-2 and *M. pneumoniae* (Table 2). Aggressive supportive care was initiated promptly, including fluid resuscitation, the correction of acidosis, intravenous glucose, fresh frozen plasma (10 mL/kg), intravenous methylprednisolone (2 mg/kg/day), and azithromycin (10 mg/kg/day).

**Table 1:** Serial laboratory findings.

Parameter	RI	D1	D2	D3	D4	D5	D6	D7	D10	D13	W2	W8
WBC (×10^9^/L)	5.5–13.6	12.84	7.72	**3.67**	6.55	9.98	8.03	**14.44**	**12.84**	**15.05**	9.05	8.90
N (×10^**9**^/L)	0.9–5.5	**10.28**	4.51	1.98	4.03	**5.85**	4.17	**6.10**	**6.61**	**7.77**	4.55	4.11
L (×10^**9**^/L)	2.7–9.1	**2.11**	2.93	**1.07**	**1.14**	**1.74**	**1.72**	5.31	4.88	6.18	3.56	4.07
Hb (g/L)	110–143	132	**104**	**105**	**107**	**99**	**97**	**98**	**89**	**86**	114	130
PLT (×10^**9**^/L)	191–596	196	**53**	**52**	**72**	**125**	221	**125**	581	**686**	404	126
CRP (mg/L)	0–8	**18**	**50**	**63**	**28**	**15**	<2	<2	<2	<2	<2	<2
Fer (ng/mL)	12–135	**10929.6**	**3889.2**	**2013.2**	**1136.2**	**895.6**	**218.0**	**207.5**	**189.5**	**281.8**	56.5	—
IL-6 (pg/mL)	0–20	**74.13**	**70.13**	**45.03**	—	—	—	—	17.96	—	8.23	—
ALT (U/L)	8–42	**17,134**	**6,792**	**4,540**	**3,065**	**1879**	**1,182**	**591**	**261**	**157**	21	40
AST (U/L)	22–59	**28,687**	**8,513**	**2,784**	**883**	**358**	**126**	**78**	43	47	42	35
FIB (g/L)	2–4	**1.76**	**1.71**	2.66	2.71	—	**4.03**	—	—	3.52	3.05	—
INR	0.88–1.16	**3.62**	**1.87**	**1.19**	1.08	—	0.98	—	—	0.98	0.89	—
D-dimer (mg/L)	0–0.55	**16.75**	**17.39**	**3.11**	**0.90**	—	**1.29**	—	—	**2.18**	—	—
LDH (U/L)	120–300	**18,615**	**5,624**	**2017**	**896**	**669**	**492**	**658**	**350**	**359**	194	230
Cr (μmol/L)	13–33	**113.5**	**179.9**	**181.5**	**163.1**	**144.5**	**105.9**	**74.5**	**33.4**	20.3	16.9	20.5
BUN (mmol/L)	1.1–5.9	**28.4**	**31.4**	**32.5**	**35.5**	**30.0**	**24.8**	**11.2**	4.4	5.3	4.3	4.1

RI, reference interval; WBC, white blood cell count; N, neutrophil count; L, lymphocyte count; Hb, hemoglobin; PLT, platelet count; CRP, C-reactive protein; Fer, ferritin; IL-6, interleukin-6; ALT, alanine aminotransferase; AST, aspartate aminotransferase; FIB, fibrinogen; INR, international normalized ratio; LDH, lactate dehydrogenase; Cr, creatinine; BUN, blood urea nitrogen. Bold values indicate parameters outside the normal reference range.

**Table 2:** Microbiological investigations.

Parameter	SARS-CoV-2	*Mycoplasma pneumoniae*
Detection method	Throat swab tNGS	Throat swab tNGS
Strain/sublineage	Omicron, JN.1 sublineage	Not typed
tNGS reads	70,475	41,299
Normalized reads	1.0 × 10^6^/100 K	1.0 × 10^6^/100 K
Estimated concentration (copies/mL)	1.0 × 10^6^	1.0 × 10^6^
Pathogenicity classification	Class A	Class A
Confirmatory testing	In-house qualitative PCR: positive	In-house qualitative PCR: positive; Serum IgM:1:80
Drug resistance gene testing	Not applicable	No macrolide resistance gene mutations detected
Criteria for active infection	Clinical presentation + high tNGS reads + positive PCR	Clinical presentation + high tNGS reads + positive PCR + positive IgM

tNGS, targeted next-generation sequencing, was performed by a clinical reference laboratory. The assay covers 225 pathogens, including adenovirus (types B, C, F, and other common types), cytomegalovirus, Epstein–Barr virus, influenza virus, and enterovirus. Except for SARS-CoV-2 and *M. pneumoniae*, all other pathogens were undetected in respiratory specimens. Normalized reads denote pathogen-specific sequences per 100 K total reads. Estimated concentration, derived via bioinformatic pipeline, is non-quantitative. Pathogenicity classification: Class A, obligate or common respiratory pathogens. Drug resistance gene testing covered the 23S rRNA gene loci A2063G, A2064G, A2067G, and C2617G of *M. pneumoniae*; no macrolide resistance-associated mutations were detected.

On hospital day 2, the patient remained hemodynamically stable, with no signs of hypoperfusion and a normal cardiac examination; the levels of high-sensitivity cardiac troponin T, N-terminal pro-B-type natriuretic peptide, electrocardiogram, and echocardiography were all normal (Figure 1). Due to persistent lethargy, a lumbar puncture was performed; the cerebrospinal fluid was clear with normal routine, biochemical, and pathogen testing (Figure 1). Laboratory investigations on days 2–3 showed rapidly declining liver enzymes, markedly improved coagulation parameters, and decreased D-dimer levels but a sharp drop in platelet count, falling hemoglobin, and progressive worsening of renal function. Urine output declined from 2–3 mL/kg/h (day 1) to 0.5–1 mL/kg/h (day 2) and fell below 0.5 mL/kg/h for 12 h on day 3, with serum creatinine peaking (Table 1). On day 3, urine microscopy showed few red blood cells (3–4/HPF), without proteinuria, red blood cell casts, or white blood cell casts. Lactate dehydrogenase, although lower than on admission, remained markedly elevated. A peripheral blood smear revealed schistocyte percentages rising from 1.5% on day 3 to 2.0% on day 4, peaking at 2.3% on day 5, and subsequently declining to 1.8% on day 6 and 0.8% by day 8 (Figure 1). These findings suggested a secondary TMA-like phenomenon. Against a background of significantly improved liver and coagulation function, low-molecular-weight heparin (enoxaparin, 0.5 mg/kg/dose subcutaneously every 12 h) was added. Urine output increased to 1–2 mL/kg/h 2 days after starting anticoagulation (days 5–6), obviating the need for hemodialysis.

A bone marrow aspirate on day 5 showed a hypercellular marrow with marked erythroid hyperplasia, normal megakaryocytes, and no hemophagocytosis. Concurrent blood tests returned normal results for soluble CD25 (sCD25) and natural killer (NK) cell activity; fasting triglyceride and fibrinogen levels did not meet the Hemophagocytic Lymphohistiocytosis 2004 (HLH-2004) diagnostic thresholds (10). The blood culture was negative; serologies for cytomegalovirus (CMV), Epstein–Barr virus (EBV), adenovirus, influenza virus, Chlamydia, Rickettsia, and respiratory syncytial virus (RSV) were all negative; the direct antiglobulin test, autoimmune hepatitis autoantibodies, and antinuclear antibodies were negative; and the serum amino acid profile and urine organic acid analysis were normal. These systematic investigations are detailed in Figure 1. The child’s condition subsequently stabilized. By day 7, urine output normalized (>2 mL/kg/h), complement C3 returned to normal, and she was transferred to the general nephrology ward. Methylprednisolone was tapered to 1 mg/kg/day intravenously and switched 3 days later to an equivalent oral dose of prednisolone (1.5 mg/kg/day). The platelet count increased substantially, serum creatinine continued to decline, and liver enzymes steadily improved, although the urine albumin-to-creatinine ratio (ACR) remained markedly abnormal (Figure 1). At discharge (day 13), the child was in good general condition without edema or hypertension; the platelet count, creatinine level, and coagulation parameters had normalized, and liver enzymes were near normal. Discharge medications included a tapering oral prednisolone regimen (1.5 mg/kg/day for 5 days, 1 mg/kg/day for 7 days, and 0.5 mg/kg/day for 7 days).

Follow-up at 2 and 8 weeks post-discharge showed persistently normal renal function and progressive normalization of ACR (Figure 1); C3 and C4 levels were normal at 2 weeks. Genetic testing results returned 2–3 weeks after discharge: family trio (proband and both parents) whole-exome sequencing (covering CFH, CFI, CD46, C3, and CFB) and whole-genome sequencing (including the CFHR1-5 region and copy number variation analysis) detected no pathogenic variants. At the 6-month follow-up, the child had no hypertension and had normal urinalysis, renal function, urinary microalbumin levels, growth, and neurodevelopment.

## Discussion

The co-detection of SARS-CoV-2 and *M. pneumoniae* has become increasingly recognized in clinical practice. One adult study reported a co-detection rate of 0–33.3% (11), and a meta-analysis found that the co-detection rate of SARS-CoV-2 with any bacterium was 7%, with *M. pneumoniae* being the most common (12). However, a systematic review including 5,867 children showed that co-detection did not significantly increase disease severity, with only one case developing a severe extrapulmonary complication (Kawasaki-like illness) (7). Case reports have demonstrated that each of these two pathogens alone can trigger a TMA-/HUS-like picture through immune-mediated mechanisms or endothelial injury (5, 6, 13). Whether the concurrent detection of both pathogens is associated with more severe systemic pathology remains unknown. To the best of our knowledge, only one case of co-detection presenting as a reactive infectious mucocutaneous eruption with mild inflammation has been reported (9), which contrasts sharply with the multisystem sequence of systemic inflammatory storm, disseminated intravascular coagulation, acute liver failure, and a secondary TMA-like phenomenon observed in our patient.

Sepsis, MIS-C, and HLH/macrophage activation syndrome (MAS) share a core feature of systemic inflammatory storm, and their clinical presentations overlap considerably in critically ill children; however, their treatment strategies and prognoses differ markedly (10, 14, 15). In the present patient, cardiac evaluations were entirely normal, ruling out MIS-C (15). When a significant decline in both platelet count and hemoglobin occurred, a bone marrow aspirate on day 5 revealed no hemophagocytosis; concurrently, soluble CD25 and NK cell activity were normal, and fasting triglyceride and fibrinogen levels did not meet HLH-2004 diagnostic thresholds (10).

Regarding the etiology of acute liver failure, the patient had no history of hepatotoxic drug or toxin exposure, and the results of the serum amino acid profiling and urine organic acid analysis were normal, preliminarily excluding drug-induced liver injury and common inherited metabolic liver diseases (16, 17). The antinuclear antibody, autoimmune hepatitis-associated autoantibodies, and direct antiglobulin test were all negative, which, together with the absence of prior liver disease, did not support a diagnosis of autoimmune hepatitis. For infectious causes, adenovirus-associated hepatitis became an important differential diagnosis following the global outbreak of severe acute hepatitis of unknown etiology in children in 2022 (17). The outsourced tNGS assay covering 225 pathogens, including common adenovirus types (B, C, and F), detected no adenovirus in the respiratory specimen, and adenovirus serology was also negative; current microbiological evidence, therefore, does not support adenovirus-associated hepatitis (17). Regarding hypoxic hepatitis, the rapid decline in aminotransferases and parallel decrease in lactate dehydrogenase are characteristic of an ischemic pattern (18), and the initial metabolic acidosis with borderline hypotension may have produced transient hepatic hypoperfusion. However, cardiac evaluations and liver imaging remained normal throughout, and hemodynamics stabilized rapidly after resuscitation. Taken together, these features suggest that transient hepatic hypoperfusion, concurrent with severe infection-induced systemic inflammation, likely contributed to the acute liver injury.

The patient’s lethargy and poor responsiveness on admission also necessitated differentiation from various encephalopathies. Head CT revealed no intracranial hemorrhage, mass, or edema. A lumbar puncture on day 2 yielded clear cerebrospinal fluid with normal routine, biochemical, and pathogen testing results, excluding intracranial infection and central nervous system vasculopathy (19). Normal blood ammonia levels, serum amino acid profiling, and urine organic acid analysis excluded metabolic encephalopathy. The clinical evolution on days 2–3 provided a critical clue: coagulation parameters had significantly improved, and D-dimer had steadily declined, indicating that DIC (20) had largely resolved. Against this background of improving coagulation, the platelet count dropped sharply, schistocytes appeared persistently on peripheral smear with progressive haptoglobin consumption, and renal function continued to deteriorate. This dissociation between recovering coagulation and worsening platelet consumption, microangiopathic hemolysis, and kidney injury cannot be explained by ongoing DIC but instead points to an independent pathological process, namely, a secondary TMA-like phenomenon (21).

Distinguishing thrombotic thrombocytopenic purpura (TTP) was also critical, as it requires urgent plasma exchange, unlike typical hemolytic uremic syndrome (HUS) (22). Normal ADAMTS13 activity definitively excluded both hereditary and acquired TTP (22). Negative stool Shiga toxin-producing *Escherichia coli* (STEC) PCR and culture excluded typical STEC-HUS (23); a negative blood culture and direct antiglobulin test excluded *Streptococcus pneumoniae*-associated HUS (24). There was no history of drug exposure, and the negative antinuclear antibody and urine organic acid analysis excluded autoimmune or metabolic TMA (25). In hereditary atypical hemolytic uremic syndrome (aHUS), complement levels are typically persistently low or require eculizumab to normalize (21). After discharge, whole-exome sequencing, whole-genome sequencing, and copy number variation analysis of the child and her parents detected no pathogenic variants, findings that do not support known variant-associated hereditary aHUS (21). The systematic differential diagnosis and its assessment are presented in Table 3, with the clinical course summarized in Figure 1.

**Table 3:** Systematic differential diagnosis.

Differential diagnosis	Supporting features	Opposing features	Assessment
Sepsis	Suspected infection (tNGS: SARS-CoV-2 + MP), organ dysfunction (hepatic, renal, coagulation), metabolic acidosis (pH 7.16, lactate 5.0 mmol/L) with PSS ≥ 2	_	**Confirmed**
MIS-C	Fever, hyperinflammation, multi-organ involvement	Normal cardiac biomarkers, ECG, and echo throughout	Excluded
HLH/MAS	Fever, Fer ≥ 500 μg/L, thrombocytopenia, anemia	No splenomegaly; bone marrow without hemophagocytosis; normal sCD25 and NK cell activity; FIB and TG not meeting HLH-2004 thresholds	Excluded
PALF	No prior liver disease, day 1: ALT 17,134 U/L, AST 28,687 U/L, INR 3.62	_	**Confirmed**
Drug-induced liver injury	Severe hepatitis	No history of hepatotoxic drug or toxin exposure	Excluded
Inherited metabolic disease	Acute liver failure in a young child	Normal serum amino acids and urine organic acids	Excluded
Autoimmune hepatitis	Severe hepatitis	Negative autoantibody workup; no prior liver disease	Excluded
AdV-associated hepatitis	Severe hepatitis; GI symptoms, post-2022 context	AdV throat swab tNGS and PCR negative; serum IgM negative	Excluded
Hypoxic/Ischemic hepatitis	Severe hepatitis, rapid aminotransferase decline, parallel LDH decrease, initial metabolic acidosis with borderline hypotension	Normal cardiac function, ECG, Echo, and liver imaging throughout; hemodynamics stabilized rapidly	**Contributing factor**
SARS-CoV-2/MP-associated hepatitis	Severe hepatitis, high tNGS reads, confirmatory PCR, positive MP IgM	_	**Contributing factor**
Secondary TMA-like phenomenon	DIC resolved; day 3: PLT 52 × 10^9^/L, Hb 105 g/L, LDH 2017 U/L, peak Cr 181.5 μmol/L; haptoglobin consumption and moderate schistocytosis	_	**Confirmed**
TTP	TMA-like phenomenon	ADAMTS13 activity normal (70%)	Excluded
STEC-HUS	TMA-like phenomenon	Stool STEC PCR and culture negative	Excluded
Pneumococcal HUS	TMA-like phenomenon	Blood culture negative; DAT negative	Excluded
Drug-induced TMA	TMA-like phenomenon	No exposure to known TMA-inducing drugs	Excluded
Hereditary aHUS	TMA-like phenomenon with low C3	C3 spontaneously normalized; WES, WGS, CNV analysis detected no pathogenic variants; anti-CFH Ab, Bb, sC5b-9 not measured	Not supported by current findings

tNGS, targeted next-generation sequencing; MP, *Mycoplasma pneumoniae*; PSS, Phoenix Sepsis Score; ECG, electrocardiography; echo, echocardiography; MIS-C, multisystem inflammatory syndrome in children; Fer, ferritin; HLH, hemophagocytic lymphohistiocytosis; MAS, macrophage activation syndrome; sCD25, soluble interleukin-2 receptor alpha; NK, natural killer; FIB, fibrinogen; TG, triglycerides; PALF, pediatric acute liver failure; ALT, alanine aminotransferase; AST, aspartate aminotransferase; INR, international normalized ratio; AdV, adenovirus; GI, gastrointestinal; PCR, polymerase chain reaction; IgM, immunoglobulin M; LDH, lactate dehydrogenase; Cr, creatinine; DIC, disseminated intravascular coagulation; PLT, platelet; Hb, hemoglobin; TMA, thrombotic microangiopathy; TTP, thrombotic thrombocytopenic purpura; HUS, hemolytic uremic syndrome; STEC, Shiga toxin-producing *Escherichia coli*; DAT, direct antiglobulin test; C3, complement component 3; WES, whole-exome sequencing; WGS, whole-genome sequencing; CNV, copy number variation; anti-CFH Ab, anti-complement factor H antibody; Bb, complement factor B breakdown product; sC5b-9, soluble terminal complement complex. Bold text indicates positive assessment results.

For the secondary TMA-like phenomenon, management focused on controlling infection, suppressing excessive inflammation, and preventing progression of microthrombi. Methylprednisolone was administered to dampen the systemic inflammatory response, and low-molecular-weight heparin was added against a background of significantly improved liver and coagulation function to prevent microthrombus progression secondary to endothelial injury. The patient ultimately achieved a full recovery with comprehensive supportive care. This study has several limitations. Anti-CFH (anti-complement factor H) antibodies and direct markers of complement alternative pathway activation (e.g., Bb fragment, sC5b-9) were not measured; therefore, the precise extent of complement alternative pathway involvement cannot be quantitatively assessed. Although whole-exome and whole-genome sequencing detected no known pathogenic variants, hereditary aHUS cannot be completely excluded given the inherent limitations of current sequencing technologies in covering deep intronic regions and unannotated variants (21).

This case suggests that in children with SARS-CoV-2 and *M. pneumoniae* co-detection who exhibit markedly elevated inflammatory markers, clinicians should be vigilant for the possibility of a systemic inflammatory storm with sequential multi-organ injury. Routine monitoring parameters are insufficient to alert clinicians to an evolving secondary TMA-like phenomenon. A surveillance protocol is recommended for high-risk children, incorporating daily platelet count, lactate dehydrogenase, peripheral blood smear for schistocyte percentage, and a urine protein-to-creatinine ratio. Of particular note, a high index of suspicion should be maintained even during the recovery phase when liver enzymes decline and coagulation improves. Once unexplained progressive thrombocytopenia and worsening renal function occur, a differential diagnostic work-up should be initiated immediately.

## Conclusion

In summary, this case documents the sequential development of acute liver failure and a secondary TMA-like phenomenon in a child with a SARS-CoV-2 and *M. pneumoniae* co-detection. The transient C3 consumption with spontaneous recovery, together with negative complement gene testing, was observed. These findings underscore the importance of vigilant monitoring for thrombotic microangiopathy in children with severe hyperinflammation and the co-detection of viral and bacterial pathogens.

## Data Availability

The original contributions presented in the study are included in the article/supplementary material, further inquiries can be directed to the corresponding author.
